# Manganese-based nanoadjuvants for enhancement of immune effect of DNA vaccines

**DOI:** 10.3389/fbioe.2022.1053872

**Published:** 2022-10-20

**Authors:** Qiang Ma, Yongxu Mu, Lidong Gong, Chuanda Zhu, Shiming Di, Ming Cheng, Jinming Gao, Jihai Shi, Liang Zhang

**Affiliations:** ^1^ Institute of Environment and Sustainable Development in Agriculture, Chinese Academy of Agricultural Sciences, Beijing, China; ^2^ College of Science, Northwest A & F University, Xianyang, China; ^3^ Department of Interventional, The First Affiliated Hospital of Baotou Medical College, Inner Mongolia University of Science and Technology, Baotou, China; ^4^ Institute of Systems Biomedicine, Department of Pharmacology, School of Basic Medical Sciences, Beijing Key Laboratory of Tumor Systems Biology, Peking University Health Science Center, Beijing, China; ^5^ College of Chemistry and Pharmacy, Northwest A & F University, Xianyang, China; ^6^ Department of Dermatology, The First Affiliated Hospital of Baotou Medical College, Inner Mongolia University of Science and Technology, Baotou, China

**Keywords:** DNA vaccine, adjuvant, H5N1 virus, manganese ion, delivery system

## Abstract

As a highly pathogenic avian influenza virus, influenza A (H5N1) has been reported to infect humans, posing a major threat to both poultry industry and public health. It is an urgent need to develop a kind of effective vaccine to prevent death and reduce the incidence rate of H5N1 avian influenza. Compared with traditional inactivated or attenuated vaccines, deoxyribonucleic (DNA) vaccines have the advantages of continuously expressing plasmid-encoded antigens and inducing humoral and cellular immunity. However, the immune effect of DNA vaccines is limited to its poor immunogenicity. Using of nanoadjuvants with DNA vaccines holds a great promise to increase the transfection efficiency and immunogenicity of DNA vaccines. In this study, we developed a nano co-delivery system with a manganese-based liposome as adjuvant for delivery of a DNA vaccine. This system has been found to protect DNA vaccine, enhance phagocytosis as well as promote activation of antigen-presenting cells (APCs) and immune cells in draining lymph nodes. In addition, the effect of this nanovaccine has been evaluated in mouse models, where it induces highly potent hemagglutination inhibitory antibody (HI) and IgG antibodies, while activating both humoral and cellular immunity in the host. Overall, this strategy opens up a new prospect for manganese nanoadjuvants in increasing the immunogenicity of DNA vaccines.

## Introduction

Influenza A (H5N1) virus also known as H5N1 virus, H5N1 avian influenza virus, is a highly pathogenic subtype of influenza A virus, with hemagglutinin (H) type 5, neuraminidase (Neuraminidase N) type1. H5N1 avian influenza virus A/goose/G Guangdong/1/96 (GS/GD/96) was first isolated from geese in Guangdong Province, China in 1996 (Sutton, 2018). Highly pathogenic influenza A (H5N1) mainly causes the death of chickens, ducks, geese and other poultry through respiratory related diseases. Investigations have shown that the H5N1 subtype of influenza virus, which is not readily transmissible to mammals, has been transmitted from birds to mammals (Kalonda et al., 2021). Mammals such as the palm cat, Indochinese cika, domestic cat and rock marten have been confirmed to be infected with the H5N1 avian influenza virus, and cases of human infection with the H5N1 avian influenza virus have been confirmed (Bui et al., 2016; Lai et al., 2016; Chen et al., 2020). Concentrated outbreaks of the H5N1 avian influenza virus in areas with high population densities pose a major threat to public health. Vaccination is one of the most effective and cost-effective interventions to prevent death and reduce the incidence of infectious pathogens (He et al., 2016). Implementing a vaccination policy can effectively reduce the susceptibility of the host to the virus while reducing the amount of virus released into the environment. Currently available vaccines against H5N1 avian influenza virus include inactivated vaccines and attenuated vaccines. Novel H5N1 avian influenza virus vaccines including mammalian cell vaccines, recombinant protein vaccines, recombinant virus-like particle (VLP) vaccines, Deoxyribonucleic (DNA) vaccines, viral vector vaccines, *etc.* have been widely explored as alternatives (Zhou et al., 2012; Kapczynski et al., 2016; Kalenik et al., 2020). DNA vaccines are considered as a potential solution for the development of effective vaccines. Compared to inactivated or attenuated vaccines, DNA vaccines are safer, easier to manufacture and store, and they can be mass-produced quickly in times of epidemic. (Kim and Samal, 2019). Despite their many advantages, only four DNA vaccines have been approved for veterinary use due to their poor immunogenicity (Davidson et al., 2005; Garver et al., 2005; Bergman et al., 2006). The reasons for the poor immunogenicity of DNA vaccines are as follows: 1) Uneven tissue distribution after DNA vaccination. 2) Naked DNA is easily degraded in tissues. 3) Naked DNA is not easily taken up by cells, resulting in low expression. 4) Lack of effective adjuvants to improve its immunogenicity (Pereira et al., 2014). Therefore, improving the delivery of DNA vaccines, protecting DNA from DNase degradation in tissues, and improving the uptake and activation of antigen-presenting cells are important issues to be solved. At the same time, effective adjuvants are also an important means to effectively improve the immunogenicity of DNA vaccines.

In recent years, a large number of studies have found that some inorganic substances and metal ions have strong physiological effects (Şerban and Enesca, 2020), such as enzymatic degradation activity (Qi et al., 2019; Zada et al., 2020), photocatalytic activity (Qi et al., 2020; Qi et al., 2022), biosensor sensor activity (Ghosh et al., 2020), *etc.* However, there are few cases of inorganic substances or metal ions as vaccine adjuvants, and only aluminum (Al) is widely recognized as a vaccine adjuvant. However, intramuscular injection of Al adjuvant can cause inflammation at the injection site and only activate the humoral immune response of the host. In recent years, it has been found that Mn^2+^ can enhance the cyclic GMP-AMP synthase-stimulator of interferon genes (cGAS-STING) signal transduction pathway (Lv et al., 2020). The cGAS-STING signal transduction pathway can effectively activate the type I interferon response and promote the release of interferon and pro-inflammatory cytokines. The cGAS-STING signal transduction pathway is considered as a bridge between innate and adaptive immunity, contributing to the maturation and migration of dendritic cells (DCs) and enhancing the cytotoxic effects of killer T cells (CTLs) and natural killer cells (NK cells) (Wang et al., 2018; Song et al., 2021). The H5N1 influenza virus has been found to suppress host immunity by suppressing the type I interferon response in infected animals (Vijayakumar et al., 2015). Therefore, Mn^2+^ has a good application prospect as an adjuvant for H5N1 influenza DNA vaccine. In this study, manganese phosphate nanoparticles with water core structure were successfully constructed, and the recombinant avian influenza virus H5N1 subtype Re-11 strain pDNA vaccine (pDNA HA Re11) was effectively loaded into the core, and its surface was covered with biocompatible lipids (pDNA HA Re11@NanoMn). The nanovaccine can effectively load and protect pDNA vaccine, enhance phagocytosis and activation of antigen presenting cells. At the same time, intracellular Mn^2+^ overload can activate the type I interferon response, which further enhances the activation of immune cells and the polarization of T cells. The results demonstrate that pDNA HA Re11@NanoMn can effectively activate the host’s cellular and humoral immune responses.

## Materials and methods

### Material

pDNA HA Re11 was designed by our research group and synthesized by Shanghai Sangon Biotechnology Co., Ltd. (2,3-Dioleoyloxy-propyl)-trimethylammonium-chloride (DOTAP) was purchased from Corden Phama. 1,2-distearoyl-sn-glycero-3-phosphoethanolamine-N- [amino (polyethylene glycol)-2000] (DSPE-PEG 2000) were purchased from Shanghai Yuanye Biotechnology. Cholesterol, 1,2-dioleoyl-sn-glycero-3-phosphate (sodium salt) (DOPA), polyoxyethylene (5) nonylphenyl ether (IGEPAL^®^ CO-520) were purchased from Sigma. Dulbecco’s modified eagle medium (DMEM), fetal bovine serum (FBS), and penicillin-streptomycin were obtained from GIBCO. Mouse anti-GAPDH monoclonal antibody, horseradish peroxidase (HRP)-labeled goat anti-mouse IgG (IgG-HRP), HRP-labeled goat anti-chicken IgG (IgG-HRP) was purchased from Invitrogen. Avian influenza virus H5 subtype (Re-11) chicken positive serum was purchased from Harbin Veken Biotechnology Co., Ltd. Mouse IFN-γ Precoated ELISA Kit, mouse IL-4 Precoated ELISA Kit, mouse IL-12 P70 Precoated ELISA Kit, mouse IL-6 Precoated ELISA Kit were obtained from Daktronics Biotechnology Co., Ltd. PE anti-mouse CD40, APC anti -mouse CD86, PE/Cyanine7 anti-mouse CD80, FITC anti-mouse CD11c, Brilliant Violet 421™ anti-mouse I-A/I-E, APC/Cyanine7 anti-mouse H-2Kd were purchased from Daktronics Biotechnology Co., Ltd. MnCl_2_•4H_2_O, Na_2_HPO_4_•12H_2_O were purchased from Sinopharm Chemical Reagent Co., Ltd. Cyclohexane, anhydrous ethanol, and chloroform (CHCl_3_) were purchased from Tongguang Fine Chemical Company. All reagents were not further processed.

## Cells and animals

Mouse bone marrow-derived dendritic cells (DC 2.4) were preserved by the Institute of Systems Biomedicine, School of Basic Medicine, Peking University School of Medicine. Chicken macrophages cells (HD 11) were obtained from the American Type Culture Collection Center (ATCC). Female BALB/c mice (6–8 weeks) and female C57BL/6n were purchased from Charles River and used for animal experiments. All animal experiments were approved by the Animal Care and Use Committee of the School of Basic Sciences, Peking University.

## Experimental method

### Construction and characterization of pDNA HA Re11@NanoMn

pDNA HA Re11@NanoMn cores and pDNA HA Re11@NanoMn were prepared according to previous methods with some adjustments (Sun et al., 2021). Briefly, anionic lipid-coated NanoMn cores were prepared by a water-in-oil microemulsion method. 300 μl of ionic solution containing 200 μg pDNA were dispersed in 15 ml of oil phase (cyclohexane/Igepal CO-520 = 79:21), stirred at room temperature for 2 h. Then 200 μl (27 mM) DOPA was added to the oil phase containing Na_2_HPO_4_ and the agitation continued for 10 min. The two oil phases containing different ions (Mn^2+^ and HPO_4_^2-^) and pDNA were mixed and continued to stir for 10 min. After adding the same amount of absolute ethanol, the precipitate was obtained by centrifugation at 15,000 *g* for 20 min and washed with absolute ethanol. NanoMn cores were dispersed in chloroform (CHCl_3_) after complete removal of absolute ethanol. 100 μl DOTAP/Chol (20 mM, V:V = 1:1) and DSPE-PEG 2000 (3 mM) were added to the solution containing NanoMn core and mixed evenly. After complete removal of CHCl_3_ by rotary evaporation, the lipid film was dispersed by gentle sonication in ddH_2_O to obtain pDNA HA Re11@NanoMn.

Particle sizing systems (PSS) was used to measure the particle size and Zeta potential of NanoMn core and pDNA HA Re11@NanoMn. To prepare TEM samples, NanoMn core and pDNA HA Re11@NanoMn were added dropwise to pure carbon copper mesh and ordinary carbon copper mesh, respectively. pDNA HA Re11@NanoMn was negatively stained with tungsten phosphate, and NanoMn core did not need negative staining. The particle size and Zeta potential were measured by dynamic light scattering (DLS). The morphology of NanoMn core and pDNA HA Re11@NanoMn was observed by transmission electron microscopy (TEM, JEOL174 1200EX).

The encapsulation efficiency of pDNA HA Re11@NanoMn was detected by fluorescence intensity method. Nanoparticles were prepared by replacing pDNA HA Re11 with FITC-labeled pDNA HA Re11. 100 μl of FITC-pDNA HA Re11@NanoMn prepared above was added to the same volume of lysis buffer (tris-HCl buffer pH 7.8, 2 mM EDTA, 0.05% Trixton 100) and incubated in a water bath at 65°C for 10 min in the darkness (Tang et al., 2015). Fluorescence intensity was detected by fluorescence spectrum analyzer (excitation wavelength 495 nm, emission wavelength 525 nm). Using different concentrations of FITC-labeled pDNA as a standard curve. The encapsulation efficiency of pDNA HA Re11@NanoMn can be calculated by the following formula: Encapsulation efficiency%= (weight of pDNA encapsulated in liposome/initial weight of pDNA) × 100%

### pH-responsive behavior and release of pDNA HA Re11@NanoMn

The buffering capacity of pDNA HA Re11@NanoMn reflects the ability of the relevant materials in the nanoparticles to absorb protons, which is relevant to promote the release of pDNA from lysosomes into the cytoplasm. The buffer capacity of the materials was determined by titration with standard sodium hydroxide (NaOH). 1ml pDNA HA Re11@NanoMn (4 μM Mn^2+^, 80 μg/ml pDNA) was added to 9 ml NaCl solution (150 mM, pH = 3). The pH value of the solution was adjusted to 9.0 with 0.1 mol/L NaOH, and the change curve of the solution pH value was recorded. NaCl solution (150 mM) was used as a negative control to calculate the buffer capacity.

To further verify the lysosomal solubilizing ability of pDNA HA Re11@NanoMn, chicken erythrocyte suspension with a volume fraction of 5% was prepared by PBS buffer with different pH values (4.0, 6.8, and 7.4). Then 100 μL pDNA HA Re11@NanoMn (4 μM, 80 μg/ml pDNA) was dispersed in the above red suspension. After incubation at 37°C for 10 min, the supernatant was removed by centrifugation at 3,000 rpm for 10 min, and the absorbance was measured using a microplate reader at 450 nm. The PBS (pH 7.4) without pDNA HA Re11@NanoMn was used as the negative control.

The *in vitro* release behavior of pDNA HA Re11@NanoMn at different pH was further evaluated. 1 mg of lyophilized FITC-pDNA HA Re11@NanoMn was dispersed in PBS (0.01 M) at pH 7.4, 6.8, and 4.5, respectively. The solution was shaken on a table concentrator at room temperature. 200 μl samples were extracted from the solution at predetermined time intervals (0 h, 2 h, 4 h, 6 h, 8 h, 10 h, 24 h, and 36 h) and filtered. Add the same volume and pH of fresh PBS to the solution. The filtered solution was used to detect the content of pDNA with a fluorescence spectrometer and draw a release curve. The concentration of Mn^2+^ was determined by inductive coupled plasma emission Spectrometer (ICP).

### Protective effect of pDNA HA Re11@NanoMn

Protective effect of nanoliposomes on pDNA HA Re11 to evaluate the stability of pDNA HA Re11 in nanoparticles. An aqueous solution containing the same concentration of pDNA HA Re11 and pDNA HA Re11@NanoMn was added to reaction buffer containing DNase I (100 mM Tris-HCl, 25 mM MgCl_2_, 1 mM CaCl_2_). The solution was incubated at 37°C for different times (0.5 h, 1 h, and 2 h). The reaction was terminated by adding 0.5 μl EDTA (200 μM) and incubating at 65°C for 10 min. After lysis of incubated pDNA HA Re11@NanoMn with lysate, free pDNA HA Re11 was detected by agarose gel method (150 V, 25 min) and observed by gel imaging system.

### Expression of pDNA HA Re11@NanoMn in DC 2.4

Western-blot was used to verify whether pDNA HA Re11@NanoMn could be expressed in cells. The specific methods refer to previous studies and make appropriate adjustments (Zhu et al., 2020). pDNA HA Re11@NanoMn was incubated with DC2.4 cells in cell culture plates for 24 h. After cell collection, lysates of about 10^5^ cells were separated by SDS- PAGE and adsorbed on PVDF membrane. After hybridization of PVDF membrane with chicken H5 specific antiserum, goat anti-chicken IgG labeled with horseradish peroxidase (HRP) was incubated to observe HA protein expression. For the internal reference test, the PVDF membrane was hybridized with monoclonal glyceraldehyde 3-phosphate dehydrogenase antibody (GAPDH), and then incubated with horseradish peroxidase (HRP) bound goat anti mouse IgG.

### Culture of mouse dendritic cells

BMDCs were cultured according to Inaba method and adjusted appropriately (Inaba et al., 1992). C57/BL6n mice (4–6 weeks) were sacrificed. The femur and tibia were removed in a sterile environment and the bone marrow was rinsed into cell culture dishes with cold PBS. The bone marrow cells were collected by centrifugation (1,600 rpm, 5 min), and the cell concentration was adjusted to 5×10^5^/ml after lysis of red blood cells. rmGM-CSF and IL-4 were added to the medium to a final concentration of 20 ng/ml, respectively. On day 3 and day 5, 0.5 ml of medium was discarded and 0.5 ml of fresh medium containing sufficient rmGM-CSF and IL-4 was added. Cells were harvested on day 6 and BMDC purity was determined by flow cytometry.

### Cytotoxicity assay of pDNA HA Re11@NanoMn

Cytotoxicity test to evaluate the safety of pDNA HA Re11@NanoMn. DC 2.4 cells and HD11 cells were seeded in 96-well plates with 5 × 10^3^ cells per well. Then, different concentrations of NanoMn (100 nM, 50 nM, 25 nM, 12.5 nM, 6.25 nM, 3.125 nM) and pDNA HA Re11@NanoMn (100 nM:200 ng, 50 nM:100 ng, 25 nM:50 ng, 12.5 nM:25 ng, 6.25 nM: 12.5 ng, 3.124 nM:6.25 ng) were dispersed into fresh medium and incubated with cells for 24 h. Cell viability was measured by Cell Counting Kit-8 (CCK-8). Briefly, 10 μl of CCK-8 solution was added to a 96-well cell culture plate, and pDNA HA Re11@NanoMn and CCK-8 solution were added to the wells without cell growth as background values. After incubation at 37°C for 2 h, the absorbance at 450 nm was detected by a microplate reader.

### Cell uptake assay

To examine cellular uptake of pDNA HA Re11@NanoMn, DC2.4 cells were cultured at a concentration of 5×10^5^/ml in glass-bottom cell culture dishes. NH_2_-modified DSPE-PEG2000 was labeled with NHS-Cy3 and then pDNA HA Re11@NanoMn was prepared. 20 μl of the above Cy3-labeled vaccine preparation was added to DC2.4 cell culture Wells. DC2.4 cells were cultured at different times (0 h, 3 h, 6 h, 12 h and 24 h). Lysosomes were labeled with LysoBlue, and the results were observed with laser confocal microscope at different times.

### Activation of BMDC cells by pDNA HA Re11@NanoMn *in vitro*

BMDC cells were adjusted to 1×10^6^/ml and cultured in 6-well plates. 20 μl of different preparations (blank liposomes, pDNA, MnCl_2_, NanoMn, pDNA HA Re11@NanoMn) were added to the cell culture Wells. PBS and LPS(1 μg) were added to blank control group and positive control group, respectively. The supernatants of cells and cells were collected after 24 h. ELISA kits were used to detect the levels of inflammatory cytokines IL-12 and IL-6 in the supernatant. After washing the cells twice with cold PBS, PE anti-mouse CD40, APC anti-mouse CD86, PE/Cyanine7 anti-mouse CD80, FITC anti-mouse CD11c, APC/Cyanine7 anti-mouse H-2KD, Brilliant Violet 421™ anti-mouse I-A/I-E were added. BMDCs were incubated for 30 min at room temperature and then the expression level of surface differentiated clusters (CD) was detected by flow cytometry. The expression level of each differentiated cluster was expressed as MFI.

### *In vivo* distribution experiment of pDNA HA Re11@NanoMn

BALB/C mice (female, 4–6 weeks) were randomly divided into two groups (*n* = 3). Free IGG and IgG-labeled pDNA HA Re11@NanoMn were injected intramuscular into the right hind leg of the mice. Mice were anesthetized with isoflurane at various time points and images were acquired in a small animal *in vivo* imaging system.

### *In vivo* animal immunization protocol

BALB/C mice (female, 4–6 weeks) were randomly divided into groups (*n* = 5), and each group of mice was intramuscular injected with different preparations (blank liposome, pDNA HA Re11, MnCl_2_, NanoMn, pDNA HA Re11@NanoMn, pDNA HA Re11@Al adjuvant). The primary immunization was performed on day 0 and the booster immunization was performed on day 14. Blood samples were collected from orbital veins of mice on days 0, 7, 14, and 28 for serum antibody detection. Spleen cells were collected and isolated on day 28 and cultured *in vitro* for cytokine detection. The experiment was carried out in accordance with the relevant experimental animal protection law.

### Hemagglutination and hemagglutination inhibition test

Hemagglutination and hemagglutination inhibition test are mainly carried out on the basis of highly pathogenic avian influenza diagnostic technology and are slightly modified according to experimental requirements. The specific method is as follows: the 4HAU antigen needs to be prepared first. PBS (25 μl) was added to the 1–12th Wells of the V-type reaction plate. The H5N1 lysing antigen solution (25 μl) was dropped into the first well, diluted to the 11th well in gradient. PBS (25 μl) and 1% chicken red blood cell suspension (25 μl) were added to each well, and the mixture was mixed evenly by shaking. After 40 min at room temperature placed observations. One hemagglutination unit (HAU) is represented by the highest dilution of the fully hemagglutinated antigen. In the hemagglutination inhibition (HI) experiment, PBS (25 μl) was added to wells 1–11th of the V-type reaction plate and 50 μl of PBS was added to the 12th well as a negative control. Immunized animal serum (25 μl) was dropped into the first well and diluted to the 10th well in a gradient sequence. 4HAU H5N1 lysis antigen solution (25 μl) was added to 1 to 10th well. 4HAU H5N1 lysis antigen solution (25 μl) was added to 1 to 11th well. After 40 min at room temperature, 25 μl of 1% chicken red blood cell suspension was added to each well. After standing at room temperature for 40 min, the V-type reaction plate was tilted to observe whether the red blood cells showed teardrop-like slide. The highest dilution of serum that completely inhibited the 4HAU antigens was taken as the HI titer.

### Detection of serum antibody levels in immunized mice by indirect ELISA

Serum anti-H5N1 avian influenza antibody IgG and subtypes IgG1 and IgG2a were detected by indirect ELISA. Enzyme-linked immunosorbent assay was performed using the method described previously with appropriate adjustments (Jesus et al., 2016). In brief, Serum from immunized animals was diluted in gradient and added to a 96-well plate coated with HA lysate protein. After 1 h of incubation, the plates were blocked with 0.5% BSA and incubated at 37°C for another 30 min. After washing with PBST, HRP-labeled IgG (1:5,000) and its subtypes HRP-IgG1 (1:2,000) and HRP-IgG2a (1:2,000) were added for incubation for 30 min. After tetramethylbenzidine (TMB) development and quenching with 2 M H_2_SO_4_, the value was read at 450 nm using a microplate reader. Serum titers were expressed as final bits. The endpoint was the last log2 dilution of an antigenic substance with an optical density (OD) at least twice higher than the control value at the same dilution. Data were normalized and balanced for variability using log2 endpoint titers before statistical analysis.

### Splenocyte cytokine production after HA restimulation

Mice were sacrificed 28 days after immunization, and spleen cells were harvested under sterile conditions to prepare spleen cell suspension. Spleen cells were counted and cultured in 24-well plates and re-stimulated with HA lysate protein. The culture supernatant of splenocytes was taken after 48 h, and the contents of IL-4 and IFN-γ cytokines in the supernatant were detected by ELISA kit. Test according to the ELISA kit manufacturer’s instructions.

### *In vivo* safety validation of pDNA HA Re11@NanoMn

The serum of each group was isolated after 28 days of immunization, and the liver function indexes (ALT and AST) and renal function indexes (CREA-J and UREA) in the serum of each group were analyzed by animal automatic biochemical analyzer. Frozen sections of major organs (heart, liver, spleen, lung, and kidney) were stained with eosin and hematoxylin (H and E) to observe the changes of tissues.

### Statistical analysis

Results are expressed as mean ± standard error of the mean (SEM). Statistical analysis was performed by GraphPad Prism 8.0.1 (GraphPad Software Inc., La Jolla, CA, United States), comparing two groups using *t*-test (normally distributed data) or Mann-Whitney test (non-normally distributed data). *p* < 0.05 were considered statistically significant.

## Results and discussion

### Preparation and characterization of pDNA HA Re11@NanoMn

**FIGURE 1:**
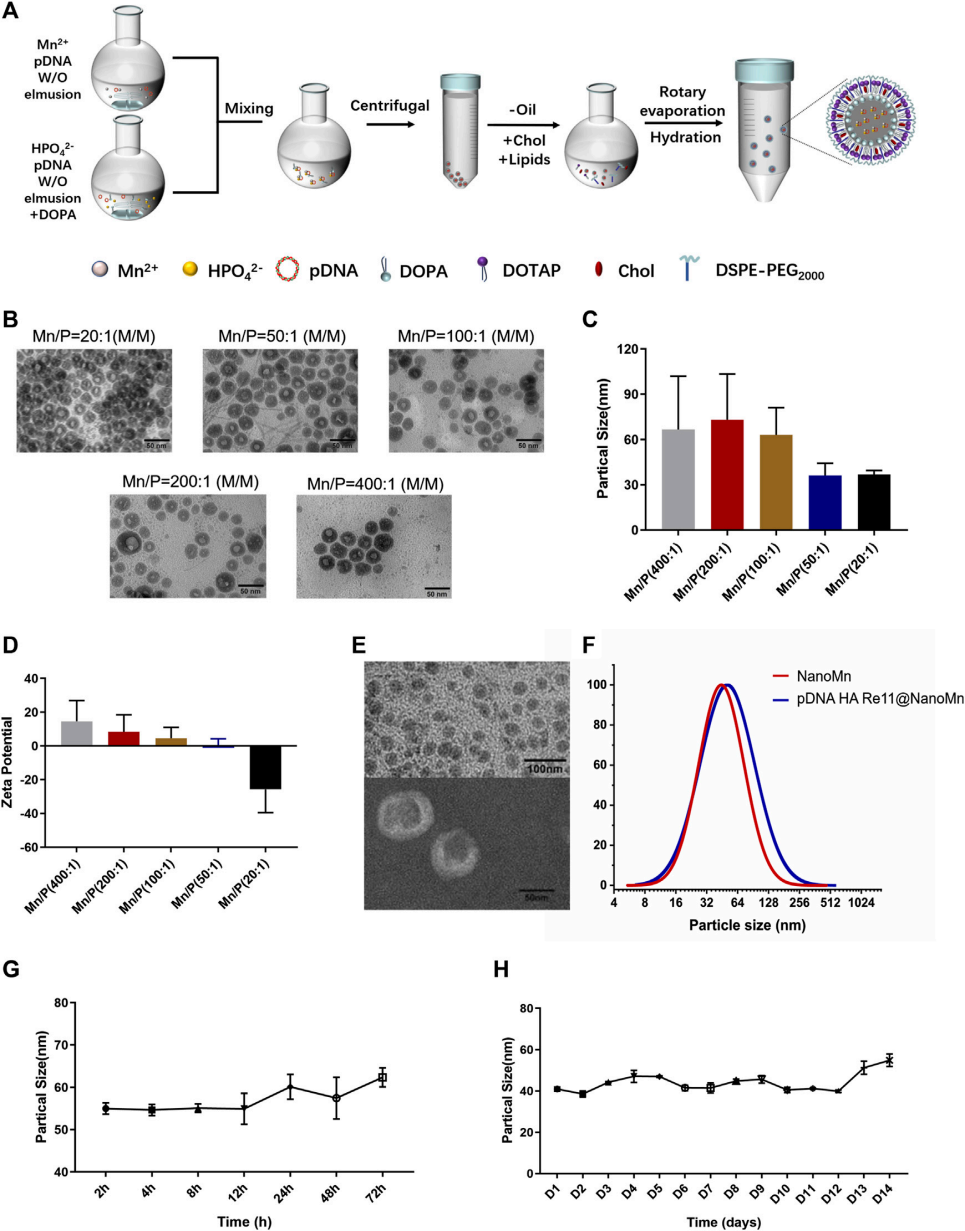
**(A)** Schematic diagram of the preparation process of pDNA HA Re11@NanoMn. **(B)** Inner core TEM image of pDNA HA Re11@NanoMn. **(C)** The particle size difference of pDNA HA Re11@NanoMn cores formed by different Mn/P molar ratios. **(D)** The Zeta potential differences of pDNA HA Re11@NanoMn cores formed by different Mn/P molar ratios. **(E)** TEM image of pDNA HA Re11@NanoMn. **(F)** Changes in particle size distribution of pDNA HA Re11@NanoMn and NanoMn. **(G)** Changes in particle size of pDNA HA Re11@NanoMn stored in PBS containing 10% FBS (37°C) for 72 h **(H)** Changes in particle size of pDNA HA Re11@NanoMn stored in PBS (4°C) for 14 days.

In this study, we designed and synthesized a manganese ion-cored, pDNA HA Re11 vaccine-loaded and lipid-coated nanoparticle using a film-ultrasonic wave dissolving technique (Figure 1A). Stable nanolipids were screened out when the molar ratio of input ions (Mn/P) was 20 in the NanoMn prepared previously (Sun et al., 2021). When pDNA needs to be loaded, the molar ratio of Mn/P needs to be appropriately increased because a large amount of phosphate in pDNA will bind to part of Mn^2+^. pDNA was loaded to prepare cores when Mn/P molar ratios were adjusted to 20, 50, and 100, respectively. The results showed that each group could form well-shaped cores. Particle size distribution and TEM images are shown in Figures 1B,C. As the Mn/P ratio decreased, the average particle size of the inner cores decreased. When the Mn/P molar ratio was 400, the particle diameter was 66.69 ± 33.24 nm, which decreased to 36.29 ± 7.63 and 36.75 ± 2.58 nm when the Mn/P molar ratio was 50 and 20, respectively. When the Mn/P molar ratio was 200 and 100, the particle size changed by 73.11 ± 28.61 nm and 63.03 ± 16.95 nm, respectively. The Zeta potential of the inner core was detected by PSS particle sizer (Figure 1D). As the Mn/P molar ratio decreases, the Zeta potential decreases gradually. The Zeta potential was positive when the Mn/P molar ratio was 400-50, and when the Mn/P molar ratio was 20, the Zeta potential was negative (−25.70 ± 13.86). According to the process constructed by NanoMn, the phosphate group in DOPA was inserted into the inner core, and the outer end was a negatively charged hydrophobic group. If excessive Mn^2+^ is added, it will be absorbed by the negative charge outside the hydrophobic group of DOPA and the Zeta potential will gradually increase. Excessive Mn^2+^ adsorption of the hydrophobic group of DOPA will affect the further assembly of DOTAP/Chol. Subsequent experiments also confirmed that a better dispersion could be formed when the molar ratio of Mn/P was 100. The size distribution and TEM of the nanoparticles are shown in Figures 1E,F. The pDNA HA Re11@NanoMn is composed of Mn^2+^ and pDNA wrapped with lipids in the inner and outer layers. Dynamic light scattering (DLS) and TEM show the particle size of pDNA HA Re11@NanoMn is about 50 nm. Figure 1E shows that the particle size of pDNA HA Re11@NanoMn is larger than that of the unloaded NanoMn, but the particle size increase is not obvious. Furthermore, the encapsulation efficiency of pDNA HA Re11@NanoMn was determined by fluorescence labeling method. The results showed that the encapsulation efficiency of pDNA HA Re11@NanoMn was 83.31% ± 2.26%. The storage stability and colloidal stability of pDNA HA Re11@NanoMn were verified for 14 days and 72 h. With the change of time (Figures 1G,H), the particle size increased slightly, but all of them were below 60 nm. These results indicate that pDNA HARe11@NanoMn has good storage stability and colloidal stability.

**FIGURE 2:**
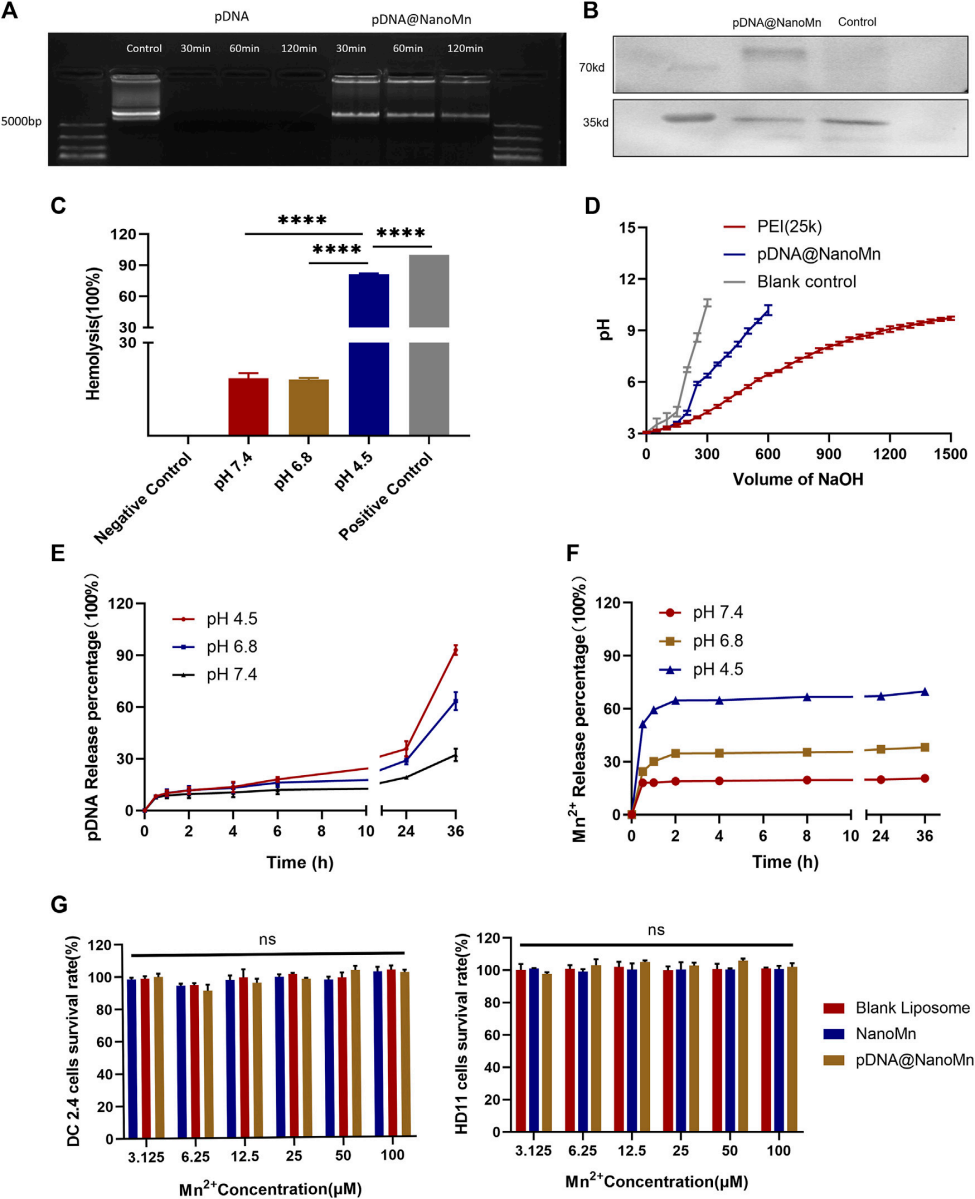
**(A)** The protective effect of pDNA HA Re11@NanoMn on pDNA HA Re11. **(B)** Western blot validation of pDNA HA Re11@NanoMn expression in DC 2.4. **(C)** The lysis of erythrocytes by pDNA HA Re11@NanoMn after incubation with erythrocytes for 1 h under physiological conditions. **(D)** Buffer capacity determination of pDNA HA Re11@NanoMn. **(E)** pDNA HA Re11@NanoMn release profile of pDNA HA Re11 in PBS buffer at different pH values (4.0,6.8, and 7.4). **(F)** Mn^2+^ release profiles of pDNA HA Re11@NanoMn in PBS buffer at different pH values (4.0,6.8, and7.4). **(G)** CCK-8 kits verify the safety of pDNA HA Re11@NanoMn and NanoMn on DC2.4 and HD11 cells.

Effectively protecting the integrity of DNA vaccines is one of the major means to improve their immunogenicity. The protective effect of nanoparticles in biologically relevant media was assessed by testing electrophoretic mobility in pDNA HA Re11. As shown in Figure 2A, the naked pDNA HA Re11 has completely disappeared after 30 min under the action of DNase I, indicating that the pDNA HA Re11 has been completely degraded by DNase I. After exposure of pDNA HA Re11@NanoMn to buffer containing DNase I for 2 h, there were obvious bright bands in the sample wells. These results suggest that pDNA HA Re11@NanoMn can inhibit the degradation of pDNA HA Re11 by DNase I in interstitial fluid or blood circulation *in vivo*. In further western blot experiments (Figure 2B), a band appeared at around 70 kd in the pDNA HA Re11@NanoMn group compared with the blank control group, indicating that the nanoparticles could effectively protect pDNA HA Re11 and deliver it into cells and successfully express antigen.

Lysosomes are important organelles for degrading substances of extracellular origin. Nanoparticles are usually degraded by endosomal lysosomal pathway after entering cells through endocytosis (Kümmel and Ungermann, 2014). To ensure that pDNA HA Re11 is not degraded during this process. pDNA HA Re11 is required to successfully detach from lysosomes, be cleaved by nanoliposomes and enter the cytoplasm. It is necessary to verify whether pDNA HA Re11@NanoMn can be released into the cytoplasm by a lysosomal escape process, resulting in antigenic cross-presentation. Previous studies have shown that erythrocyte membranes can well mimic lysosomal membranes (Banerjee et al., 2012). Chicken erythrocyte membrane was selected to simulate lysosomal membrane. PBS solutions with different pH values (4.5, 6.8, and 7.4) were selected to simulate lysosomal environment, intracellular environment and extracellular environment, respectively. The results (Figure 2C) showed that the hemolysis rate of pDNA HA Re11@NanoMn was less than 20% at pH 7.4 and 6.8, indicating that the nanoparticles had good biocompatibility at physiological pH. When pH was 4.5, the nanoparticles had obvious hemolysis and the hemolysis rate was more than 80%. The hemolysis of pDNA HA Re11@NanoMn under acidic conditions may be related to the swelling and cleavage of pDNA HA Re11@NanoMn under acidic conditions. The lipid material contained in the nanoparticles after swelling has a high buffer capacity and absorb more H^+^, thus increasing the pH value of red blood cells and leading to the inflow of a large amount of Cl^+^ and H_2_O. Eventually leading to the rupture of red blood cells. This process is similar to that of lysosomal escape. It can be predicted that the nanoparticles have the ability to promote the escape of pDNA HA Re11 from the lysosome and release into the cytoplasm in the acidic environment of the lysosome.

The proton sponge effect is one of the important mechanisms for the escape of nanoparticles from lysosomes (Lee et al., 2021). The buffering capacity of pDNA HA Re11@NanoMn was further evaluated to indirectly assess its proton sponge effect. The results showed that the buffering capacity of pDNA HA Re11@NanoMn was significantly higher than that of NaCl solution (negative control), but it was significantly lower than that of PEI (positive control) (Figure 2D). The strong buffering capacity of PEI leads to severe lysosomal rupture and ultimately to host cell death. This is also the main reason that causes high toxicity of PEI and limits its clinical application. The lower buffer capacity of pDNA HA Re11@NanoMn compared with PEI ensures lysosomal escape of nanoparticles under biosafe conditions. In addition, we investigated the release behavior of pDNA HA Re11@NanoMn in PBS buffer at different pH values (Figures 2E,F) and showed that the release behavior of nanoparticles was significantly enhanced in acidic solution compared with neutral solution, which is consistent with our previous study (De Koker et al., 2011). In this process, the release of Mn^2+^ was significantly faster than that of pDNA. It is assumed that a large number of Mn^2+^ bind to the outer layer of pDNA during the formation of the inner core. Therefore, when the nanoparticles were cleaved under acidic conditions, the release of Mn^2+^ was significantly faster than that of pDNA.

### pDNA HA Re11@NanoMn Cytotoxicity test

The standard CCK-8 cell viability assay was used to test the safety of NanoMn and pDNA HA Re11@NanoMn on HD11 and DC2.4. As shown in Figure 2G, the effects of NanoMn and pDNA HA Re11@NanoMn on DC2.4 cells and HD11 cells at different concentrations of Mn^2+^ and pDNA HA Re11 were not significantly toxic compared with the control group. The results showed that pDNA HA Re11@NanoMn has good biosafety.

### Cellular uptake assays

**FIGURE 3:**
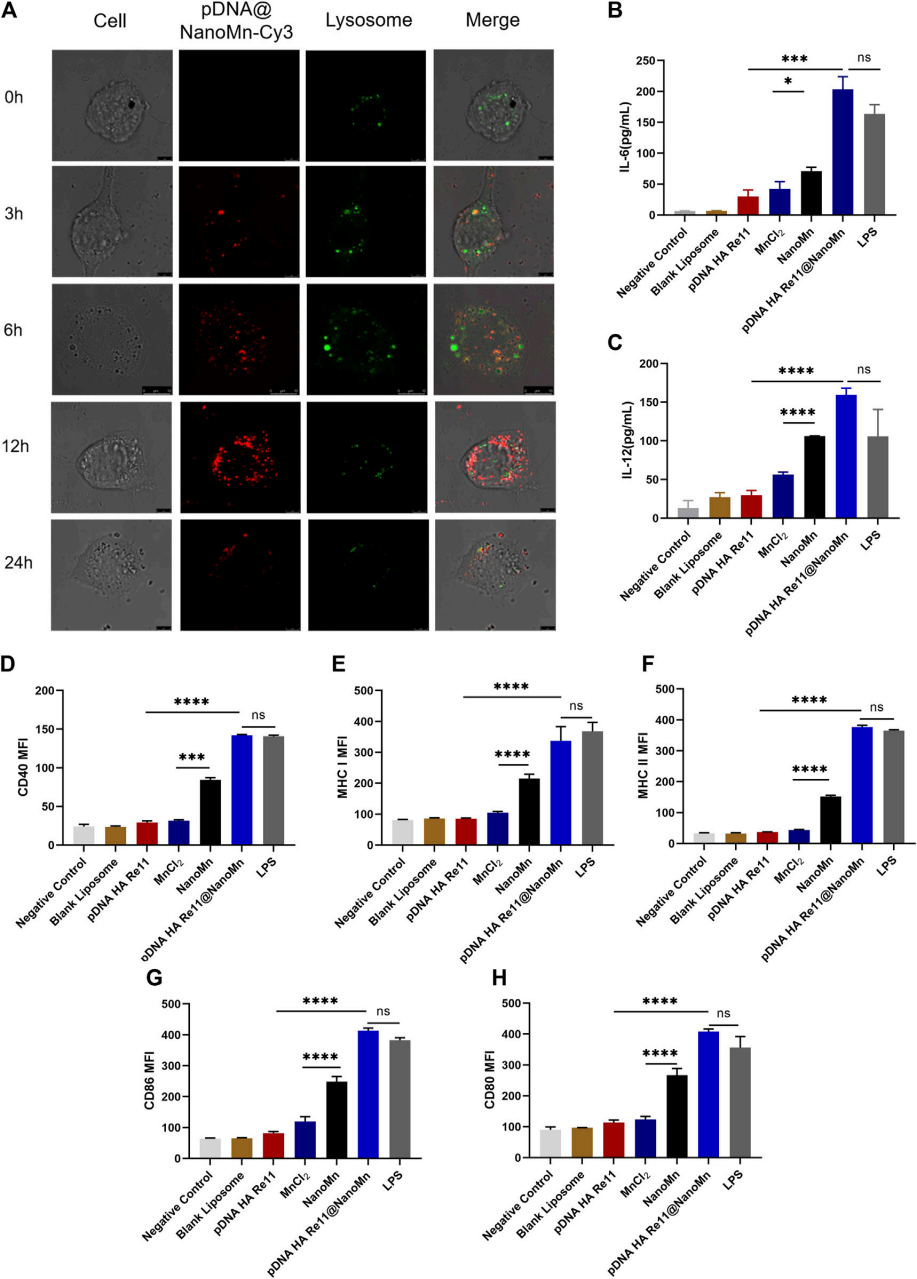
**(A)** *In vitro* cellular uptake assay. Intracellular distribution images of DC2.4 cells incubated with Cy3-labeled pDNA HA Re11@NanoMn for 24 h, respectively. **(B,C)**.Different preparations stimulated dendritic cells to release cytokines IL-6 **(B)** and IL-12 **(C)** levels. d-h. Effects of different preparations on dendritic cell activation. Dendritic cell surface CD40 **(D)** MHC I **(E)**, MHC II **(F)** CD86 **(G)** and CD80 **(H)** expression levels.

Uptake of DNA vaccines by antigen presenting cells (APC) is one of the most important steps in achieving immunogenicity. Observation by laser confocal microscope at different time points. As shown in Figure 3A, with the prolongation of time, the red fluorescence of Cy3 in DC2.4 cells gradually increased. The strongest red fluorescence of Cy3 was observed at 12 h, indicating that the cell endocytosis of pDNA HA Re11@NanoMn was enhanced. A significant decrease in intracellular fluorescence at 24 h implied that pDNA HA Re11@NanoMn had been degraded within lysosomes and pDNA HA Re11 had been released into the cytoplasm. During this process, there is a clear overlap between the red fluorescence of Cy3 and the green fluorescence of labeled lysosomes. This phenomenon suggests that liposomes are transported to lysosomes by endocytosis. The liposomal sponge effect of pDNA HA Re11@NanoMn can efficiently escape pDNA HA Re11 into the cytoplasm, which is the basis for antigen cross-presentation.

### Activation of dendritic cells (BMDC) by pDNA HA Re11@NanoMn *in vitro*.

BMDCs are usually quiescent *in vivo* and change from quiescent to activated in response to antigen or related cytokine stimulation. BMDCs engulf antigens and move from the injection site through the lymphatic system to nearby lymph nodes, where they differentiate into mature BMDCs. Major histocompatibility complex I (MHC I) and major histocompatibility complex II (MHC II) are abundantly expressed on the surface of mature BMDC. At the same time, the expression levels of CD80, CD86, and CD40 were increased. CD80 binds to CD28 receptors on naive T cells and provides a costimulatory signal for T cell activation. The binding of CD40 and CD40L facilitates the activation of stimulated B cells and promotes the differentiation of B cells into antibody-secreting plasma cells and memory B cells. Activated BMDCs in lymphoid tissues bind to relevant receptors on the surface of T cells through costimulatory molecules such as CD80, CD86 and CD40, and secrete related cytokines such as IL-12 and IL-6 to regulate the differentiation of T cells. Activated BMDCs present antigens to CD^4+^ T cells *via* the MHC II pathway to activate the host’s humoral immunity, and present the antigens to CD^8+^ T cells *via* the MHC I pathway to activate the host’s cellular immunity. Activated BMDCs also secrete cytokines such as IL-6 and IL-12. IL-6 activates lymphocytes and promotes the production of antibodies, promoting Th2 immune responses and causing the host’s humoral immune response. IL-12 induce T cells and NK cells to secrete IFN-γ, promote the Th1 immune response and cause the host’s cellular immune response (De Koker et al., 2011). The secretion levels of IL-6 and IL-12 in BMDCs were detected by Elisa kit, and the results were shown in Figures 3B,C. The secretion of IL-6 and IL-12 in the pDNA HA Re11@NanoMn group was significantly higher than the other groups. Compared with the blank control group, the secretion of IL-6 and IL-12 in MnCl_2_ group and NanoMn group was also significantly improved. Further flow cytometry showed (Figures 3D–H) that the expression of MHC I on the surface of BMDC closely corresponded to IL-6 and IL-12 in the pDNA HA Re11@NanoMn group. In addition, the expression level of MHC II in pDNA HA Re11@NanoMn group was significantly higher than that in other groups except LPS. These results indicate that pDNA HA Re11@NanoMn can effectively activate BMDCs and enhance the cross-presentation ability of BMDCs, which lays a foundation for subsequent induction of cellular and humoral immune responses simultaneously. Compared with pDNA HA Re11, the expression of costimulatory molecules on the surface of BMDCs was significantly increased in the pDNA HA Re11@NanoMn group. In addition, it can be observed from the results that the expressions of CD80, CD86, and CD40 in the NanoMn group were also significantly higher than the MnCl_2_ group, which corresponded to the results of cytokine secretion level. We speculate that NanoMn entry into the cytoplasm through endocytosis causes Mn^2+^ to achieve intracellular overload more easily than MnCl_2_, thus maximizing the effect of Mn^2+^ as an adjuvant. Previous studies have shown that Mn^2+^ activates the innate immune system *in vivo* by activating the cGAS-STING pathway. The cGAS-STING signaling pathway plays a key role in host immune response and host defense. The activation of cGAS-STING signaling pathway can induce the production of type I interferons and proinflammatory cytokines, and then promote the maturation of antigen presenting cells and the differentiation and activation of CD^8+^T cells. pDNA HA Re11@NanoMn enters BMDCs by endocytosis, pDNA HA Re11 generates corresponding antigen in cytoplasm through transcription and translation process, and then is presented to BMDCs surface by MHC I to activate CD^8+^ T cells. We can see that pDNA HA Re11@NanoMn can significantly activate BMDCs and stimulate their maturation to promote antigen cross-presentation.

### Inhibition of hemagglutination induced by pDNA HA Re11@NanoMn *in vivo*

**FIGURE 4:**
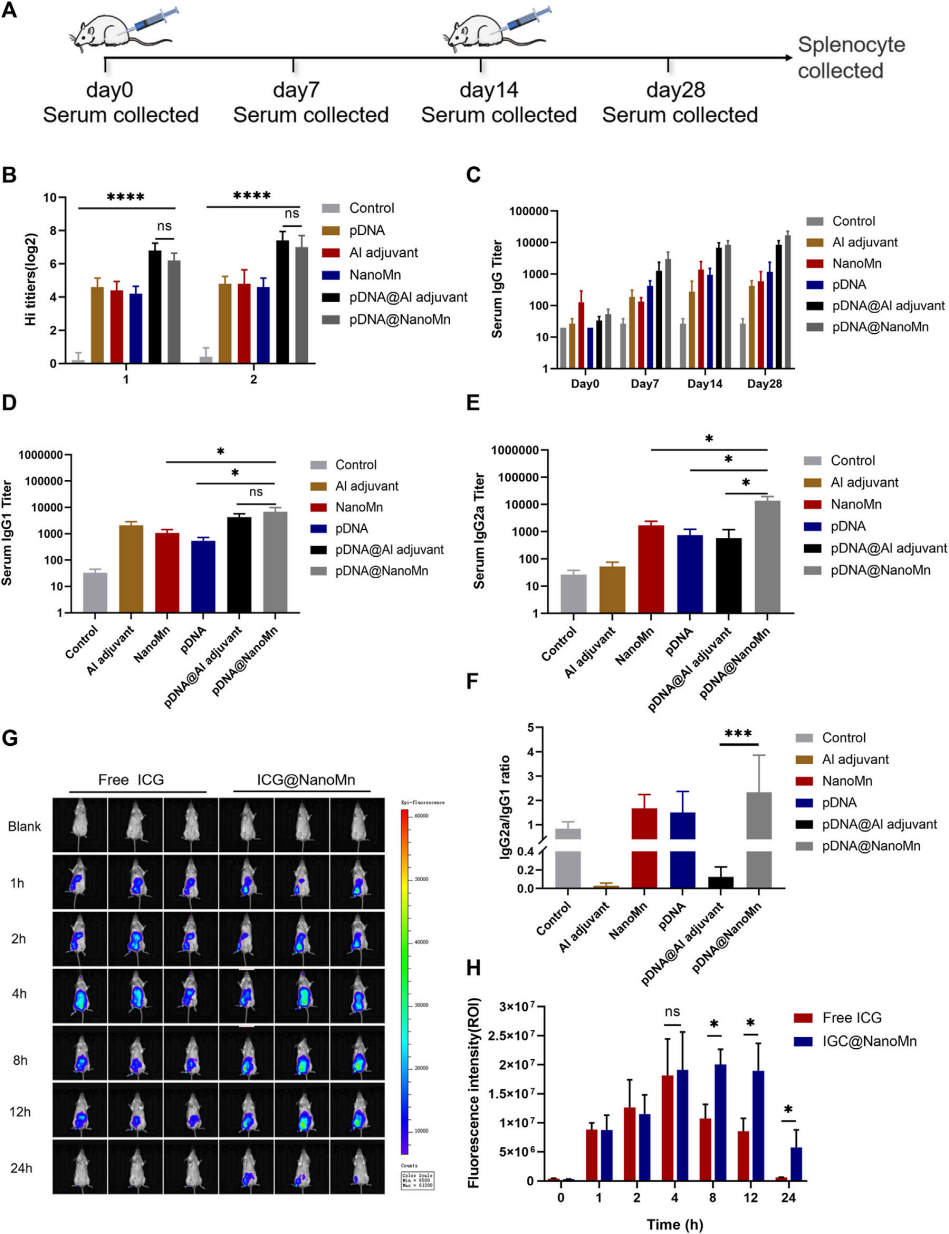
**(A)** Schematic diagram of the mouse immunization protocol. **(B)** HI antibody titers on the 14th and 28th days after immunization. **(C)** IgG antibody titers in serum at different time points **(D–E)** Serum IgG2 and IgG1 titers on day 28 after immunization. **(F)** Ratio of IgGI to IgG2a. **(G)** *In vivo* imaging of small animals at different time points. **(H)** Quantitative analysis of fluorescence intensity at injection sites at different time points.

Viruses containing hemagglutinin can agglutinate human or animal red blood cells, and the corresponding antibodies can inhibit the phenomenon of hemagglutination. Determination of HI antibody titer in serum samples is an important indicator of antibody response to intramuscular vaccine preparations. Blood samples were collected from orbital veins of mice on day 7 after two immunizations for hemagglutination inhibition(Figure 4A). The results of the hemagglutination inhibition (HI) test (Figure 4B) showed that the HI antibody titers induced by the pDNA HA Re11@NanoMn group were significantly higher than those induced by the other groups except the Al adjuvant group on the seventh day after both immunizations. This indicates that pDNA HA Re11@NanoMn can induce strong hemagglutination inhibition.

### pDNA HA Re11@NanoMn induced changes in serum antibodies and cytokines *in vivo*

From results of specific IgG antibody titer in serum (Figure 4C), it can be seen that the IgG antibody titer induced by pDNA HA Re11@NanoMn was significantly higher than the other groups. The differences between cellular and humoral immune responses to H5N1 antigen induced by pDNA HA Re11@NanoMn were further analyzed. We compared the titers of two subtypes of IgG antibodies (IgG1 and IgG2a). In each group. Th1-type cytokines stimulate the synthesis of IgG2a antibodies and assist cellular immune responses. Th2-type cytokines stimulate the synthesis of IgG1 antibodies, which mainly mediate humoral immune responses. The changes of IgG1 and IgG2a antibody expression were compared to reflect the changes of Th1 and Th2 cells, and then reflect the level of cellular and humoral immune responses. (Ma et al., 2012). The results showed that the levels of IgG1 and IgG2a antibodies in the pDNA HA Re11@NanoMn group were significantly higher than the other groups, indicating that the pDNA HA Re11@NanoMn group could induce both humoral and cellular immunity (Figures 4D,E). The expression level of IgG1 induced by Al adjuvant was significantly higher than IgG2a, suggesting that Al adjuvant tended to induce humoral immune response, which was consistent with the results of previous studies. From the specificity analysis, there was no significant difference in IgG1 antibody titer in the pDNA HA Re11@NanoMn group compared with the pDNA HA Re11@Al adjuvant group, but there was a significant difference in IgG2a antibody titer. Thus, pDNA HA Re11@NanoMn could induce a more balanced mixed Th1/Th2 type immune response, whereas pDNA HA Re11@Al adjuvant group was more likely to induce a Th2 type humoral immune response. In the ratio of IgG2a to IgG1 (Figure 4F), it can be seen that the pDNA HA Re@Al adjuvant group was significantly lower than the pDNA HA Re@NanoMn group. The results showed that pDNA HA Re@NanoMn group could induce a more balanced Th1/Th2 mixed immune response, while pDNA HA Re@Al adjuvant group was more likely to induce humoral immune response. The results showed that nanocarriers could effectively protect pDNA HA Re11 and efficiently deliver it into cells. After transcription and translation into HA Re11 protein in the cytoplasm, the cross-presentation of antigen is realized, and finally the synchronous response of cellular and humoral immunity is realized.

One of the important roles of adjuvants is the antigen depot effect, which can prolong the interaction time between antigens and the immune system and improve the immune effect of the host (Riehl et al., 2018). To investigate whether NanoMn as an antigen carrier has an antigen depot effect, we encapsulated a water-soluble fluorescent indocyanine blue-green dye (ICG) in the water core of NanoMn. Free ICG was used as a control group. The results showed (Figures 4G,H) that the fluorescence intensity of the free ICG group began to decrease significantly after 8 h of injection, and it was difficult to detect the fluorescence at the injection site after 24 h. The fluorescence intensity of the ICG@NanoMn group was similar to free ICG group at 8 h. But the fluorescence intensity of the ICG@NanoMn group was significantly higher than that of free ICG group after 8 h, and fluorescence was still detectable at the injection site at 24 h. The results showed that the ICG@NanoMn group prolonged the residence time of antigen at the injection site and had a better antigen depot effect.

### *In vivo* safety testing of pDNA HA Re11@NanoMn

**FIGURE 5:**
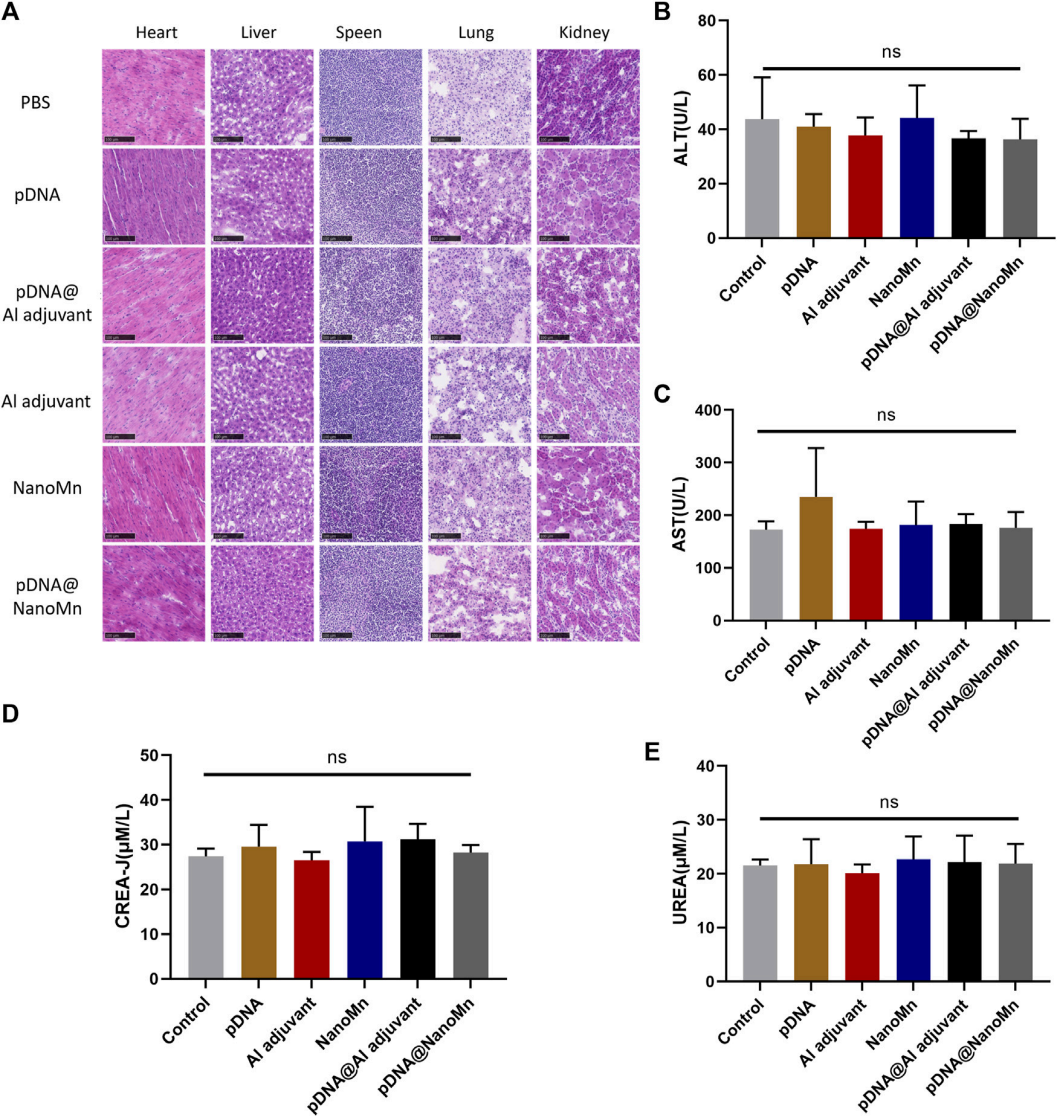
*In vivo* safety verification of pDNA HA Re11@NanoMn. **(A)** H&E staining of mouse vital organs (heart, liver, spleen, lung, and kidney) 28 days after immunization with different formulations. **(B,C)** Changes of liver function indexes (AST, ALT) in blood biochemistry of mice after immunization with different preparations for 28 days **(D,E)** Changes of renal function indexes (CREA-J, UREA) in blood biochemistry of mice after immunization with different preparations for 28 days.

Biochemical analysis was performed on the serum of immunized mice. Compared with the blank control group, there was no significant difference in the liver function function-related indexes (AST and ALT) and renal function-related indexes (CREA-J and UREA) of the pDNA HA Re11@NanoMn administration group. (Figures 5B–E). H&E staining results of tissue sections of important organs (heart, liver, spleen, lung, and kidney) were shown (Figure 5A). Compared with the blank control group, no obvious pathological changes were found in the pDNA HA Re11@NanoMn administration group. According to the above data, pDNA HA Re11@NanoMn has good biocompatibility and can be predicted to be safe for clinical use.

## Conclusion

In conclusion, we designed a nanoliposome (pDNA HA Re11@NanoMn) co-delivered with H5N1 DNA vaccine using Mn^2+^ as an adjuvant. Such nanoliposomes can effectively achieve protective DNA vaccines, enhance phagocytosis and activation of APCs, which in turn activate immune cells in draining lymph nodes and polarize T cell subsets. *In vivo* experiments confirmed that pDNA HA Re11@NanoMn could induce high antigen-specific antibody titers and virus-specific HAI, effectively activating the host’s cellular and humoral immune responses. In addition, Mn^2+^ and lipid components are easy to obtain and have the advantages of safety and good biocompatibility. This is critical for emergency preparedness and safety regulation to address pandemic emergencies.

## Data Availability

The original contributions presented in the study are included in the article/supplementary material, further inquiries can be directed to the corresponding authors.

## References

[B1] Banerjee S. Sen K. Pal T. K. Guha S. K. (2012). Poly(styrene-co-maleic acid)-based pH-sensitive liposomes mediate cytosolic delivery of drugs for enhanced cancer chemotherapy. Int. J. Pharm. X. 436 (1-2), 786–797. 10.1016/j.ijpharm.2012.07.059 22884831

[B2] Bergman P. J. Camps-Palau M. A. McKnight J. A. Leibman N. F. Craft D. M. Leung C. (2006). Development of a xenogeneic DNA vaccine program for canine malignant melanoma at the Animal Medical Center. Vaccine 24 (21), 4582–4585. 10.1016/j.vaccine.2005.08.027 16188351

[B3] Bui C. Bethmont A. Chughtai A. A. Gardner L. Sarkar S. Hassan S. (2016). A systematic review of the comparative epidemiology of avian and human influenza A H5N1 and H7N9 - lessons and unanswered questions. Transbound. Emerg. Dis. 63 (6), 602–620. 10.1111/tbed.12327 25644240

[B4] Chen X. Wang W. Wang Y. Lai S. Yang J. Cowling B. J. (2020). Serological evidence of human infections with highly pathogenic avian influenza A(H5N1) virus: A systematic review and meta-analysis. BMC Med. 18 (1), 377. 10.1186/s12916-020-01836-y 33261599 PMC7709391

[B5] Davidson A. H. Traub-Dargatz J. L. Rodeheaver R. M. Ostlund E. N. Pedersen D. D. Moorhead R. G. (2005). Immunologic responses to West Nile virus in vaccinated and clinically affected horses. J. Am. Vet. Med. Assoc. 226 (2), 240–245. 10.2460/javma.2005.226.240 15706975

[B6] De Koker S. Lambrecht B. N. Willart M. A. van Kooyk Y. Grooten J. Vervaet C. (2011). Designing polymeric particles for antigen delivery. Chem. Soc. Rev. 40 (1), 320–339. 10.1039/b914943k 21060941

[B7] Garver K. A. LaPatra S. E. Kurath G. (2005). Efficacy of an infectious hematopoietic necrosis (IHN) virus DNA vaccine in Chinook *Oncorhynchus tshawytscha* and sockeye *O. nerka* salmon. Dis. Aquat. Organ. 64 (1), 13–22. 10.3354/dao064013 15900683

[B8] Ghosh S. Chen Y. George A. Dutta M. Stroscio M. A. (2020). Fluorescence resonant Energy transfer-based quantum dot sensorfor the detection of calcium ions. Front. Chem. 8, 594. 10.3389/fchem.2020.00594 32903607 PMC7438717

[B9] He F. Leyrer S. Kwang J. (2016). Strategies towards universal pandemic influenza vaccines. Expert Rev. Vaccines 15 (2), 215–225. 10.1586/14760584.2016.1115352 26641724

[B10] Inaba K. Inaba M. Romani N. Aya H. Deguchi M. Ikehara S. (1992). Generation of large numbers of dendritic cells from mouse bone marrow cultures supplemented with granulocyte/macrophage colony-stimulating factor. J. Exp. Med. 176 (6), 1693–1702. 10.1084/jem.176.6.1693 1460426 PMC2119469

[B11] Jesus S. Soares E. Costa J. Borchard G. Borges O. (2016). Immune response elicited by an intranasally delivered HBsAg low-dose adsorbed to poly-ε-caprolactone based nanoparticles. Int. J. Pharm. X. 504 (1-2), 59–69. 10.1016/j.ijpharm.2016.03.013 26976502

[B12] Kalenik B. M. Góra-Sochacka A. Stachyra A. Olszewska-Tomczyk M. Fogtman A. Sawicka R. (2020). Response to a DNA vaccine against the H5N1 virus depending on the chicken line and number of doses. Virol. J. 17 (1), 66. 10.1186/s12985-020-01335-9 32381003 PMC7206725

[B13] Kalonda A. Phonera M. Saasa N. Kajihara M. Sutcliffe C. G. Sawa H. (2021). Influenza A and D viruses in non-human mammalian hosts in africa: A systematic review and meta-analysis. Viruses 13 (12), 2411. 10.3390/v13122411 34960680 PMC8706448

[B14] Kapczynski D. R. Tumpey T. M. Hidajat R. Zsak A. Chrzastek K. Tretyakova I. (2016). Vaccination with virus-like particles containing H5 antigens from three H5N1 clades protects chickens from H5N1 and H5N8 influenza viruses. Vaccine 34 (13), 1575–1581. 10.1016/j.vaccine.2016.02.011 26868083 PMC4794445

[B15] Kim S. H. Samal S. K. (2019). Innovation in newcastle disease virus vectored avian influenza vaccines. Viruses 11 (3), 300. 10.3390/v11030300 PMC6466292 30917500

[B16] Kümmel D. Ungermann C. (2014). Principles of membrane tethering and fusion in endosome and lysosome biogenesis. Curr. Opin. Cell. Biol. 29, 61–66. 10.1016/j.ceb.2014.04.007 24813801

[B17] Lai S. Qin Y. Cowling B. J. Ren X. Wardrop N. A. Gilbert M. (2016). Global epidemiology of avian influenza A H5N1 virus infection in humans, 1997-2015: A systematic review of individual case data. Lancet Infect. Dis. 16 (7), e108–e118. 10.1016/s1473-3099(16)00153-5 27211899 PMC4933299

[B18] Lee J. Sands I. Zhang W. Zhou L. Chen Y. (2021). DNA-inspired nanomaterials for enhanced endosomal escape. Proc. Natl. Acad. Sci. U. S. A. 118 (19), e2104511118. 10.1073/pnas.2104511118 33941681 PMC8126792

[B19] Lv M. Chen M. Zhang R. Zhang W. Wang C. Zhang Y. (2020). Manganese is critical for antitumor immune responses via cGAS-STING and improves the efficacy of clinical immunotherapy. Cell. Res. 30 (11), 966–979. 10.1038/s41422-020-00395-4 32839553 PMC7785004

[B20] Ma M. Wang L. Yang J. Cai H. Shi J. Zhang S. (2012). Age-related impaired Th1 responses to PRV vaccine *in vivo* in aged pigs. Mol. Immunol. 52 (3-4), 217–223. 10.1016/j.molimm.2012.05.016 22750068

[B21] Pereira V. B. Zurita-Turk M. Saraiva T. D. L. De Castro C. P. Souza B. M. Mancha Agresti P. (2014). DNA vaccines approach: From concepts to applications. World J. Vaccines 4 (2), 50–71. 10.4236/wjv.2014.42008

[B22] Qi K. Li Y. Xie Y. Liu S. Y. Zheng K. Chen Z. (2019). Ag loading enhanced photocatalytic activity of g-C3N4 porous nanosheets for decomposition of organic pollutants. Front. Chem. 7, 91. 10.3389/fchem.2019.00091 31001509 PMC6454074

[B23] Qi K. Lv W. Khan I. Liu S. (2020). Photocatalytic H2 generation via CoP quantum-dot-modified g-C3N4 synthesized by electroless plating. Chin. J. Catal. 41 (1), 114–121. 10.1016/s1872-2067(19)63459-5

[B24] Qi K. Zhuang C. Zhang M. Gholami P. Khataee A. (2022). Sonochemical synthesis of photocatalysts and their applications. J. Mat. Sci. Technol. 123, 243–256. 10.1016/j.jmst.2022.02.019

[B25] Riehl M. Harms M. Lucas H. Ebensen T. Guzmán C. A. Mäder K. (2018). Dual dye *in-vivo* imaging of differentially charged PLGA carriers reveals antigen-depot effect, leading to improved immune responses in preclinical models. Eur. J. Pharm. Sci. 117, 88–97. 10.1016/j.ejps.2018.01.040 29408551

[B26] Şerban I. Enesca A. (2020). Metal oxides-based semiconductors for biosensors applications. Front. Chem. 8, 354. 10.3389/fchem.2020.00354 32509722 PMC7248172

[B27] Song Y. Liu Y. Teo H. Y. Hanafi Z. B. Mei Y. Zhu Y. (2021). Manganese enhances the antitumor function of CD8^+^ T cells by inducing type I interferon production. Cell. Mol. Immunol. 18 (6), 1571–1574. 10.1038/s41423-020-00524-4 32801367 PMC8166851

[B28] Sun Y. Yin Y. Gong L. Liang Z. Zhu C. Ren C. (2021). Manganese nanodepot augments host immune response against coronavirus. Nano Res. 14 (5), 1260–1272. 10.1007/s12274-020-3243-5 33391623 PMC7770383

[B29] Sutton T. C. (2018). The pandemic threat of emerging H5 and H7 avian influenza viruses. Viruses 10 (9), 461. 10.3390/v10090461 PMC6164301 30154345

[B30] Tang J. Li L. Howard C. B. Mahler S. M. Huang L. Xu Z. P. (2015). Preparation of optimized lipid-coated calcium phosphate nanoparticles for enhanced *in vitro* gene delivery to breast cancer cells. J. Mat. Chem. B 3 (33), 6805–6812. 10.1039/c5tb00912j PMC4869335 27213045

[B31] Vijayakumar P. Mishra A. Ranaware P. B. Kolte A. P. Kulkarni D. D. Burt D. W. (2015). Analysis of the crow lung transcriptome in response to infection with highly pathogenic H5N1 avian influenza virus. Gene 559 (1), 77–85. 10.1016/j.gene.2015.01.016 25592823

[B32] Wang C. Guan Y. Lv M. Zhang R. Guo Z. Wei X. (2018). Manganese increases the sensitivity of the cGAS-STING pathway for double-stranded DNA and is required for the host defense against DNA viruses. Immunity 48 (4), 675–687.e7. e7. 10.1016/j.immuni.2018.03.017 29653696

[B33] Zada A. Khan M. Qureshi M. N. Liu S. Y. Wang R. (2020). Accelerating photocatalytic hydrogen production and pollutant degradation by functionalizing g-C3N4 with SnO2. Front. Chem. 7, 941. 10.3389/fchem.2019.00941 32133336 PMC7039856

[B34] Zhou F. Zhou J. Ma L. Song S. Zhang X. Li W. (2012). High-yield production of a stable Vero cell-based vaccine candidate against the highly pathogenic avian influenza virus H5N1. Biochem. Biophys. Res. Commun. 421 (4), 850–854. 10.1016/j.bbrc.2012.04.101 22554519

[B35] Zhu N. Wang D. Xie F. Qin M. Lin Z. Wang Y. (2020). Fabrication and characterization of calcium-phosphate lipid system for potential dental application. Front. Chem. 8, 161. 10.3389/fchem.2020.00161 32269987 PMC7111464

